# Few-Shot Induction of Generalized Logical Concepts via Human Guidance

**DOI:** 10.3389/frobt.2020.00122

**Published:** 2020-11-18

**Authors:** Mayukh Das, Nandini Ramanan, Janardhan Rao Doppa, Sriraam Natarajan

**Affiliations:** ^1^Device Intelligence, Samsung R&D Institute India - Bangalore, Device Intelligence, Bangalore, India; ^2^Erik Jonsson School of Engineering and Computer Science (ECS), The University of Texas at Dallas, ECS, Dallas, TX, United States; ^3^School of Electrical Engineering & Computer Science (EECS), Washington State University, EECS, Pullman, WA, United States

**Keywords:** cognitive systems, logics for knowledge representation, relational learning, knowledge representation and reasoning, human in the loop (HITL)

## Abstract

We consider the problem of learning generalized first-order representations of concepts from a small number of examples. We augment an inductive logic programming learner with 2 novel contributions. First, we define a distance measure between candidate concept representations that improves the efficiency of search for target concept and generalization. Second, we leverage richer human inputs in the form of advice to improve the sample efficiency of learning. We prove that the proposed distance measure is semantically valid and use that to derive a PAC bound. Our experiments on diverse learning tasks demonstrate both the effectiveness and efficiency of our approach.

## 1. Introduction

We study the case of learning from *few examples*, of which *one-shot* learning is a special case (Lake et al., 2015). We consider a challenging setting—that of learning explainable, decomposable, and generalizable (first-order) concepts from few examples. Plan induction becomes a special case where a generalizable plan is induced from a single (noise-free) demonstration. As an example, consider building a tower that requires learning *L-shapes* as a primitive. In our formulation, the goal is to learn a *L-shape* from a single demonstration. Subsequently, using this concept, the agent can learn to build a rectangular base (with 2 *L-shapes*) from another single demonstration and so on till the tower is fully built. Concept learning has been considered as problem solving by reflection (Stroulia and Goel, 1994), mechanical compositional concepts (Wilson and Latombe, 1994), learning probabilistic programs (Lake et al., 2015), etc. While successful, they are not considered in one-shot learning except with SVM (Tax, 2001), or with a neural network (Kozerawski and Turk, 2018).

Our work has two key differences. First, we aim to learn an “easily interpretable,” “explainable,” “decomposable,” and “generalizable” concepts as first-order Horn clauses (Horn, 1951) (which are akin to If-Then rules). Second, and perhaps most important, we “do not assume the existence of a simulator (for plans) or employ a closed-world assumption” to generate negative examples. Inspired by Mitchell's (1997) observation of futility of bias-free learning, we employ domain expertise as inductive bias. The principle of structural risk minimization (Vapnik, 1999) shows how optimal generalization from extremely sparse observations is quite difficult. The problem is difficult in structured domains since most relations are false. Thus, few-shot induction of generalized logical concepts is challenging. We employ iterative revision of first-order horn clause theories using a novel scoring metric and guidance from a human. *We emulate a “student” who learns a generalized concept from an example provided by the “teacher,” by both reflecting as well as asking relevant questions*.

We propose *Guided One-shot Concept Induction* (Goci) for learning in relational domains^1^. Goci builds upon an inductive logic program (ILP) learner (Muggleton, 1991) with two key extensions. First, a modified scoring function that explicitly computes distances between concept representations. We show the relation to *Normalized Compression Distance* (NCD) for plan induction settings. Consequently, we demonstrate that NCD is a valid distance metric. Second, we use domain knowledge from human expert as inductive bias. Unlike many advice taking systems that employ domain knowledge before training, Goci identifies the relevant regions of the concept representation space and *actively solicits guidance* from the human expert to find the target concept in a sample-efficient manner. Overall, these two modifications allow for more effective and efficient learning using Goci that we demonstrate both theoretically and empirically.

We make the following key contributions:

1. We derive a new distance-penalized scoring function that computes definitional distances between concepts, henceforth termed as “conceptual distance.”
2. We treat the human advice as an inductive bias to accelerate learning. Our ILP learner actively solicits richer information from the human experts than mere labels.
3. Our theoretical analyses of Goci prove that (a) our metric is indeed a valid distance, and (b) NCD between plans is a special case of our metric.
4. We show a PAC analysis of the learning algorithm based on Kolmogorov complexity.
5. We demonstrate the exponential gains in both sample efficiency and effectiveness of Goci on diverse concept induction tasks with one or a few examples.

## 2. Background and Related Work

Our approach to **Concept Learning** is closely related to Stroulia and Goel (1994)'s work, which learns logical problem-solving concepts by reflection. Goci's scoring metric is more general and applicable to both concepts and plans and can be used for learning from a few examples. While we use discrete spatial structures as motivating examples, Goci is not limited to discrete spaces, similar to Wilson and Latombe (1994)'s work. Goci is also related in spirit to probabilistic (Bayesian) program induction for learning decomposable visual concepts (Lake et al., 2015), which illustrates how exploiting decomposability is more effective. Our approach leverages not only decomposability but also implicit relational structure.

### 2.1. One/Few-Shot Learning and Theory Induction

Our problem setting differs from the above in that it requires learning from sparse examples (possibly one). Lake et al. (2015) propose a one-shot version of Bayesian program induction of visual concepts. There is also substantial work on one/few-shot learning (both deep and shallow) in a traditional classification setting (Bart and Ullman, 2005; Vinyals et al., 2016; Wang et al., 2018), most of which either pre-train with gold-standard support example set or sample synthetic observations. We make no such assumptions about synthetic examples. ILP (Muggleton, 1991) inductively learns a logical program (first-order theory) that covers most of the positive examples and few of the negative examples by effectively employing background knowledge as search bias. In concept learning, generalization is typically performed as a search through space of candidate inductive hypotheses by (1) structuring, (2) searching, and (3) constraining the space of theories. FOIL (Quinlan, 1990) is an early noninteractive learner with the disadvantage that it occasionally prunes some uncovered hypotheses. This is alleviated in systems like FOCL by introducing language bias in the form of user-defined constraints (Pazzani, 1992). With *Interactive ILP*, learner could pose questions and elicit expert advice that allows pruning large parts of search space (Sammut and Banerji, 1986; Rouveirol, 1992). To incorporate new incoming information, ILP systems with *theory revision* incrementally refine and correct the induced theory (Sammut and Banerji, 1986; Muggleton, 1988). While Goci is conceptually similar to ALEPH (Srinivasan, 2007), it learns from a few examples and actively acquires domain knowledge by interacting with human expert incrementally.

### 2.2. Knowledge-Guided Learning

Background knowledge in ILP is primarily used as search bias. Although the earliest form of knowledge injection can be found in explanation-based approaches (Shavlik and Towell, 1989), our work relates to preference-elicitation framework (Braziunas and Boutilier, 2006), which guides learning via human preferences as an inductive bias. Augmented learning with domain knowledge as an inductive bias has long been explored across various modeling formalisms, including traditional machine learning (Fung et al., 2003), probabilistic logic (Odom et al., 2015), and planning (Das et al., 2018). Our human-guided Goci learner aims to extend these directions in the context of learning generalizable complex concepts from a few examples(including plans). Similar problem setting of concept learning from incomplete/sparse observations has also been explored in the cognitive science paradigm via explanation-based inductive program synthesis (Flener, 1997; Kitzelmann and Schmid, 2006).

The idea of augmented learning with human guidance/knowledge has also been extensively studied in the context of evolutionary computation. Interactive evolutionary systems (Eiben and Smith, 2015) use expert guidance to emulate a holistic fitness function that would otherwise depend on a very restricted pre-defined fitness model. The potential richness of such knowledge can be leveraged in not just evolutionary parent selection but can also optimize other parameters that leads to faster convergence, especially in mutations (Wendt et al., 2010). ILP has been shown to be conceptually similar to mutative EA in the context of program induction (Wong and Leung, 1997) and hence knowledge-guided mutations are related to knowledge augmented search in ILP. Thus, in our problem setting, the interaction module that seeks human guidance to select the most useful constraints (detailed in section 3.2.3) is similar in spirit to interactive (knowledge guided) evolutionary mutation process. However, our underlying search strategy and optimization is based on ILP.

## 3. Guided One-Shot Concept Induction

We are inspired by a teacher (human) and student (machine) setting in which a small number of demonstrations are used to learn generalized concepts (Chick, 2007). Intuitively, the description provided by a human teacher tends to be modular (can have distinct logical partitions), structured (entities and relations between them), and in terms of known concepts. Hence, a vectorized representation of examples is insufficient. We choose a logical representation, specifically a “function-free restricted form of first-order logic (FOL)” that models structured spaces faithfully.

**Given**: A set of “facts” or assertions, that is, a set of ground literals (or trajectories) describing 1 (or few) instance(s) of an unknown concept, availability of an expert to provide guidance and a knowledge-base of known concepts.

**To Do:** Learning a representation, by inducing a first-order logic program, of the given concept that optimally generalizes the given instance(s) effectively.

The input to Goci is the description of the instances(s) of a concept that the human teacher provides. An example is, thus, conjunction of a set of ground literals (assertions). The output of Goci is a least general generalization (LGG) horn clause from the *input* example(s).

### 3.1. Concept Representation

**Figure 1:**
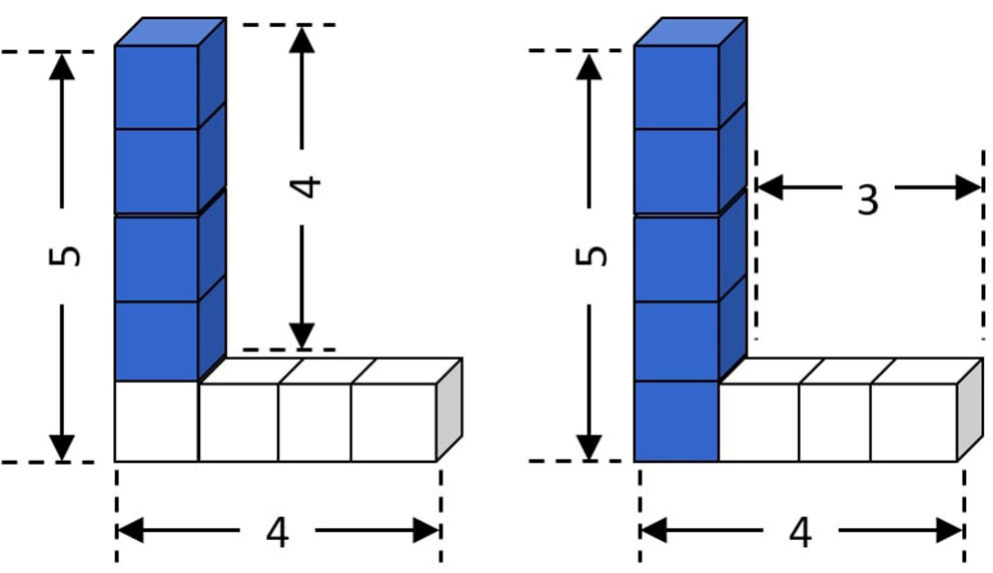
Concept 𝕃 (*base* = 4, *height* = 5), described as composition of a **Tower** and a **Row**.

Consider the following example input to the Goci framework. The input here is an instance of the structural concept *L* (illustrated in Figure 1).

**Example 1**. *An instance in a minecraft domain can be a* 𝕃 *with dimensions height* = 5, *base* = 4 *(Figure 1)*. 𝕃(*S*), *Height*(*S*, 5), *Base*(*S*, 4), *s is the concept identifier and may be described as conjunction of ground literals*,


Row(A)∧   Tower(B)∧   Width(A, 4)∧  Height(S, 5)∧
Base(S, 4)∧    Contains(S, A)∧    Contains(S, B)∧
Height(B, 4)∧ SpRel(B, A,′NWTop′),


*which denotes* 𝕃 *as composition of a “Row” of w* = 4 *and a “Tower” of h* = 4 *with appropriate literals describing the scenario (Figure 1, left). As a special case, under partial or total ordering assumptions among the ground literals, an input instance can represent a plan demonstration*.

We aim to learn the optimally generalized (decomposable) representation of the concept (𝕃 in the context of the aforementioned example) referred by the one/few instances that were passed to Goci as input. Before further discussion on the learning such a generalized (decomposable) representation let us first define formally what a concept representation signifies in our setting.

**Definition 1**. *Concepts in* Goci *are represented as horn clause theories. A theory T is defined as*, $T=C\left(s^{k}\ldots \right):-\vee \left[\wedge_{i=1}^{N}f_{i}\left(t_{1},\ldots ,t_{j}\right)\right]$, *where the body* $\wedge_{i=1}^{N}f_{i}\left(t_{1},\ldots ,t_{j}\right)$ *is a conjunction of literals indicating known concepts or relationships among them, such that any* $t_{j}\in V\cup \left\{s^{k}\right\}\cup C$ *where V is the set of all logical variables in the clause, C is the set of constants in the domain of any logical variable. The head* $C\left(s^{k}\ldots \right)$ *identifies a target concept, and the terms* {*s*^*k*^} *are logical variables that denote the parameters of the concept assuming there are k* = {1, …, *K*} *parameters including the identifier to the given instance of the concept. Since a concept can be described in multiple ways (Figure 1), the final theory will be a disjunction over clause bodies with the same head. A (partial) instantiation of a theory T is denoted as T/θ*.

Note that these definitions allow for the reuse of concepts, potentially in a hierarchical fashion. We believe that this is *crucial in achieving human-agent collaboration*.

**Example 2**. *Figure 1 illustrates an instance of the concept* 𝕃 *that can be described in multiple ways. A possible one is*,

$$\begin{array}{l} 𝕃\left(s\right):-[Height\left(s,h_{s}\right),Base\left(s,w_{s}\right),Contains\left(s,a\right),\\ \;Contains\left(s,b\right),Row\left(a\right),Tower\left(b\right),\\ \;Width\left(a,w_{a}\right),Height\left(b,h_{b}\right),Equal\left(w_{s},w_{a}\right),\\ \;Sub\left(h_{b},h_{s},1\right),SpRel\left(b,a{,}^{\prime \prime }NWTop^{\prime \prime }\right)]\\ \;\vee \;[Height\left(s,h_{s}\right),Base\left(s,w_{s}\right),Contains\left(s,a\right),\\ \;Contains\left(s,b\right),Row\left(a\right),Tower\left(b\right),\\ \;Width\left(a,w_{a}\right),Height\left(b,h_{b}\right),Equal\left(h_{s},h_{b}\right),\\ \;Sub\left(w_{a},w_{s},1\right),SpRel\left(b,s{,}^{\prime \prime }W^{\prime \prime }\right)] \end{array}$$

The generalization must be noted. The last argument of the SpRel() is a constant, as only this particular spatial alignment is appropriate for the concept of the 𝕃 structure. Although the input is a single instance (Example 1), Goci should learn a generalized representation such as Example 2. Another interesting aspect is the additional constraints: Equal(X,Y) and Sub(X,Y,N). While such predicates are a part of the language, they are not typically described directly in the input examples. However, they are key to generalization, since they express complex interactions between numerical (or non-numerical) parameters. Also note that the head predicate of the clause could have been designed differently as per Definition 1. For instance, in case of Example 2, the head predicate could have folded in the dimensional parameters—𝕃(*s, h*_*s*_, *w*_*s*_). However, the number of such dimensional parameters can vary across different concepts. Hence to maintain generality of representation format during implementation, we push the dimensional parameters of the learnable concept into the body of the clause.

A specific case of our concept learning (horn clause induction) framework could be plan induction from sparse demonstrations. This can be achieved by specifying time as the last argument of both the state and action predicates. Following this definition, we can allow for plan induction as shown in our experiments. Our novel conceptual distance is clearer and more intuitive in the case of plans as can be seen later.

**Definition 2** (Decomposable:). *A concept* C *is decomposable if it is expressed as a conjunction of other concepts, and one or more additional literals to model the interactions*. $C⇐\left(\wedge_{i}{C\prime }_{i}\right)\wedge \left(\wedge_{j}B_{j}\right)$. *Here* ${C\prime }_{i}$ *are literals that represent other concepts that are already present in the knowledge base of the learner and B*_*j*_ *are literals that either describe the attributes of* ${C\prime }_{i}$ *or interactions between them*.

Decomposable allows for an unknown concept to be constructed as a composition of other known concepts. Goci learns the class of decomposable concepts since it is intuitive for the “human teacher” to describe. Decomposable concepts faithfully capture the *modular* and *structured* aspect of how humans would understand and describe instances. It also allows for a hierarchical construction of plans.

**Example 3**. *Following the Minecraft structure described in 2, note how* 𝕃 *is described with already known concepts* ${C\prime }_{1}=Row()$ *and* ${C\prime }_{2}=Tower()$ *and the other literals such as Height*(*b, h*_*b*_), *SpRel*(*b, a*, ”*NWTop”*), … ∈ {*B*_*j*_}, *that is, they describe the parameters of the known concepts or interactions between them. Note that known concepts in the knowledge base could have been manually coded in by experts or learned previously and are essentially represented in the same way. For instance, Row() can be encoded as recursive the clause program representing a composition of one block and one unit shorter row*,

$$\begin{array}{l} Row(r):-[Width(r,w_{r}),Block(a),Row(b),Width(b,w_{b}),\\ \;SpRel(a,b,\prime \prime Eas\prime \prime ),Sub(w_{b},w_{r},1)]\\ \;\vee [Width(r,w_{r}),Equal(w_{r},1),Block(a)] \end{array}$$

*Tower() could also be defined in the knowledge base in the same way. When the optimally general representation of the concept* 𝕃 *is learned that is persisted in the knowledge base as well, such that more complex concepts can be represented by decomposing into* 𝕃 *and other known concepts*.

An obvious question that arises here is why $\left\{B_{j}\right\}⊈\left\{C^{\prime }\right\}$? that is, why can the other literals not be treated similarly as a part of the knowledge base of known concepts? Ideally, that would be correct. However, that will also cause infinite levels of concept definitions, which cannot be implemented in practice. Additionally, following the paradigm of a student–teacher scenario, it can always be assumed that the student has prior understanding of many concepts from outside the current system. Thus, we can safely assume, without loss of generality, that set of literals {*B*_*j*_} are implicitly understood and defined as a part of the framework itself. This argument applies to the semantics of the “constraint predicates” (described later) as well.

Finally, before we discuss the details of the learning methodology, let us briefly look into a motivating, and presently relevant, real-world scenario that represents our problem setting.

**Figure 2:**
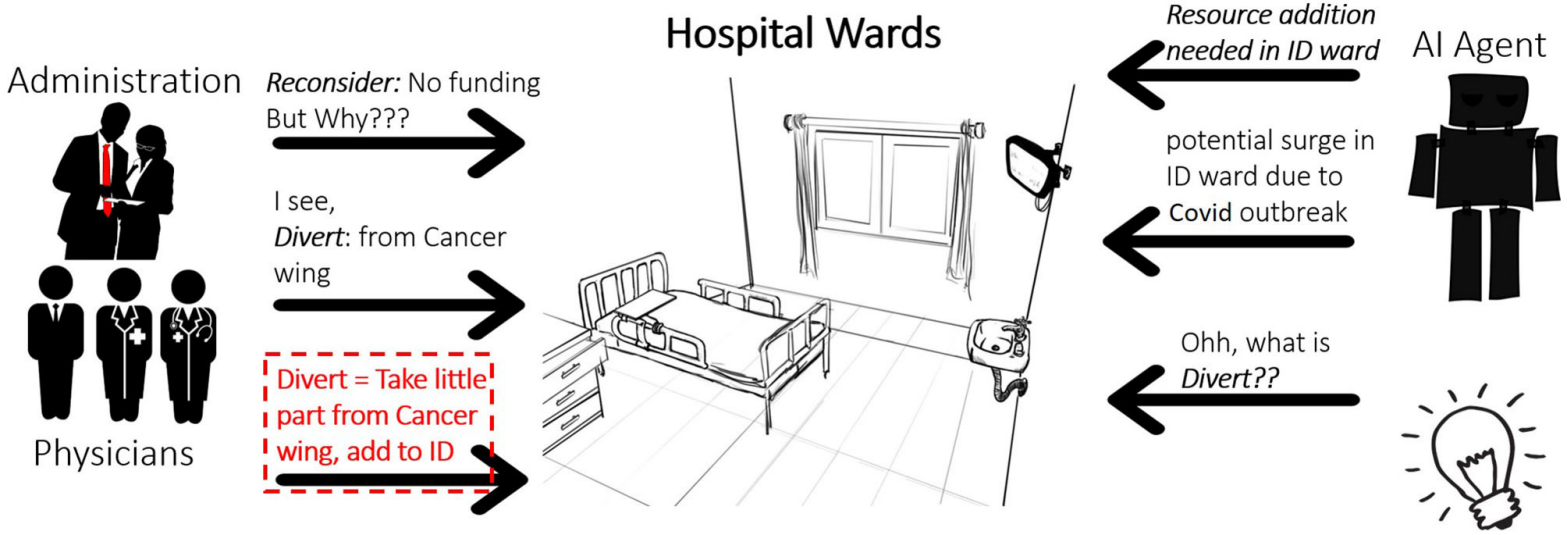
A motivating real-world scenario for concept induction. The concept learnt by the AI agent is “Divert()”.

**Example 4**. *Consider a decision support AI system for resource planning and management in hospitals as illustrated in Figure 2. The AI agent forecasts the need for increased resources in the infectious diseases (ID) ward, given the early signs of an outbreak of some disease such as Covid-19 or Ebola, and a potential spike in ID ward admissions. However, as noted by the administrators and/or physicians there is not enough budget to procure additional resources for ID ward. But the problem is quite critical and needs to be solved. So the human teacher (administrators in this case) teaches the AI agent the concept of “Divert” -ing resources from Cancer ward since cancer ward admissions are usually stable and does not have spikes. The AI agent is hence expected to learn a generalized representation of the concept of divert such that it may be applied later for other wards or for other tasks and furthermore in a “decomposable” fashion. For instance, “Divert” may be learned as a clause program such as*,

$$\begin{array}{l} Divert\left(R,qty_{R}\right):-To\left(R,Loc_{dest}\right),AcquireFrom\\ \;\left(R,Loc_{source}^{1},qty_{1},\right),\\ \;AcquireFrom\left(R,Loc_{source}^{2},qty_{2}\right),\\ \;AssignTo\left(R,Loc_{dest},qty_{dest}\right),\\ \;sum\left(qty_{1},qty_{2},qty_{dest}\right) \end{array}$$

*Obviously, the above representation assumes that concepts such as “AcquireFrom()” are known concepts, either implicitly defined inside the learning framework or its explicit representation has been learned and persisted inside the knowledge base in the past*.

The above example is solely to motivate the potential impact of our problem setting and the proposed solution. For an explanation of different components and aspects of Goci, we refer to the much simpler and unambiguous structural example outlines earlier (𝕃).

### 3.2. Methodology

#### 3.2.1. Search

ILP systems perform a greedy search through the space of possible theories. Space is typically defined, declaratively, by a set of mode definitions (Muggleton, 1995) that guide the search. We start with the most specific clause (known as a bottom clause) (Srinivasan, 2007) from the ground assertions and successively add/modify literals that might improve a rule that best explains the domain. Typically, the best theory is the one that covers the most positive and least negative examples. Thus, it optimizes the likelihood of a theory *T* based on the data. We start with a bottom clause and variablize the statements via anti-substitution. Variabilization of *T* is denoted by θ^−1^ = {*a*/*x*}, where *a* ∈ *consts*(*T*), *x* ∉ *vars*(*T*). That is, anti-substitution θ^−1^ is a mapping from occurrences of ground terms in *T* to new or existing logical variables.

**Evaluation Score:** We redesign the ILP scoring (e.g., ALEPH's compression heuristics) as:

- The user-provided advice forces the learner to learn longer theory, hence the *search space can be exponentially large*. Thus, modes alone are not sufficient as the search bias.
- There is only one (a few) positive training example(s) to learn from and *many possible rules can accurately match the training example*. Coverage-based scores fail.

Most learners optimize some form of likelihood. For a candidate theory *T*, likelihood given data 𝔻 is *LL*(*T*) = log*P*(𝔻|*T*) (i.e., coverage). To elaborate further, in most classification tasks in discrete domains (with categorical/ordinal feature and target variables), goodness of fit of candidate models is achieved via the measure of how well the candidate models explain (or cover) the given data, that is, a good model is the one that will predict positive class for maximum possible positive examples and for minimum possible negative examples. This measure is expressed as likelihood of the data given a candidate model. In Goci, we have one (at most few) positive example(s). Coverage will not suffice. Hence, we define a modified objective as follows.

(1) $$\begin{array}{l} T^{*}=\underset{T\in \tau }{\text{arg}\text{ min}}\left(-LL\left(T\right)+D\left(T/\theta_{X},X\right)\right) \end{array}$$

where T^*^ is the optimal theory, τ is the set of all candidate theories, and *D* is the conceptual distance between the instantiated candidate theory *T*/θ_*X*_ and the original example *X*. Recall that a theory 𝕋 is a disjunction of horn clause bodies (or conjunction of clauses).

#### 3.2.2. Distance Metric

Conceptual distance, *D*(*T*/θ_*X*_, *X*), is a penalty in our objective. The key idea is that any learned first-order horn clause theory must recover the given instance by equivalent substitution. However, syntactic measures, such as edit distance, are not sufficient since changing even a *single literal*, especially, literals that indicate interconcept relations, could potentially result in a completely different concept. For instance, in blocks-world, the difference between a block being in the middle of a row and one at the end of the row can be encoded by changing one literal. Hence, a more sophisticated semantic distance such as conceptual distance is necessary (Friend et al., 2018). However, such distances require deeper understanding of the domain and its structure.

Our solution is to employ *interplan distances*. Recall that the concepts Goci can induce are decomposable and, hence, are equivalent to parameterized planning tasks. One of our key contributions is to exploit this equivalence by using a domain-independent planner to find grounded *plans* for both the theory learned at a particular iteration *i*, *T*_*i*_ and the instance given as input, *X*. We then compute the normalized compression distance (NCD) between the plans.

**NCD:** Goldman and Kuter (2015) proved that NCD is arguably the most robust interplan distance metric. NCD is a reasonable approximation of *Normalized Information Distance*, which is not computable (Vitányi et al., 2009). Let the plans for *T*_*i*_/θ_*X*_ and *X* be π_*T*_ and π_*X*_. To obtain NCD, we execute string compression (lossy or lossless) on each of the plans as well as the concatenation of the two plans to recover the compressed strings *C*_*T*_, *C*_*X*_, and *C*_*T,X*_, respectively. NCD between the plans can be computed as,

(2) $$\begin{array}{l} NCD\left(\pi_{T},\pi_{X}\right)=\frac{C_{T,X}-\min\left(C_{T},C_{X}\right)}{\max\left(C_{T},C_{X}\right)} \end{array}$$

**Figure 3:**
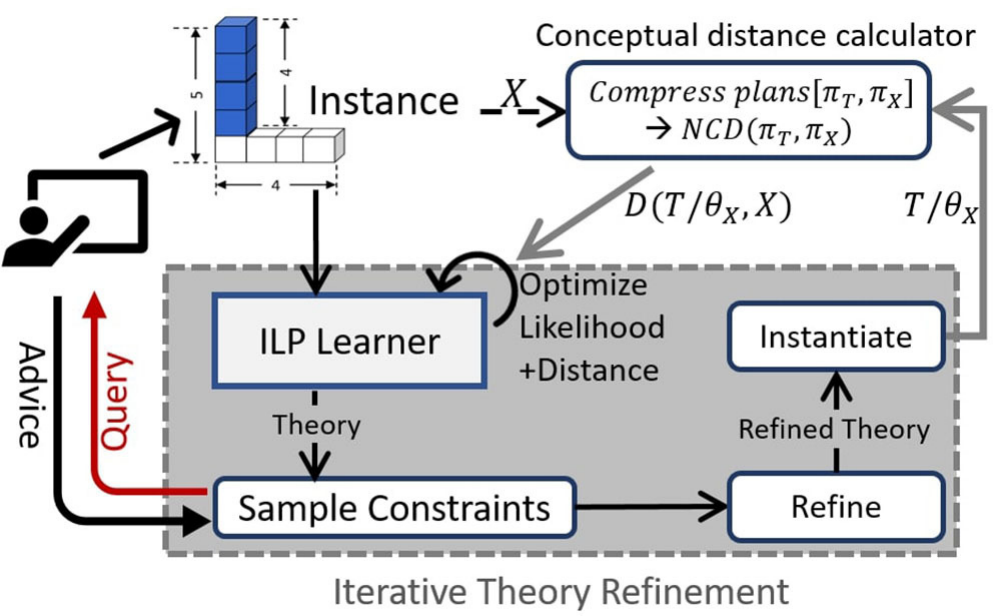
Highlevel overview of our Guided One-shot Concept Induction (Goci) framework.

The conceptual distance between a theory *T* and *X* is the NCD between the respective plans, *D*(*T*/θ_*X*_, *X*) = *NCD*(π_*T*_, π_*X*_). This entire computation is performed by the *conceptual distance calculator* as shown in Figure 3.

**Observations**: **(1)** Conceptual distance as a penalty term in the *LL* score ensures that the learned theory will correctly recover the given example/demonstration. **(2)** *D*(*T*/θ_*X*_, *X*) generalizes to the *Kolmogorov–Smirnov* statistic between two target distributions if we induce probabilistic logic theories. We prove these insights theoretically.

#### 3.2.3. Human Guidance

The search space in ILP is provably infinite. Typically, language bias (modes) and model assumptions (closed world) are used to prune the search space. However, it is still intractable with one (or few) examples. So, we employ human expert guidance as constraints that can directly refine an induced theory, acting as a strong inductive bias. Also, we are learning decomposable concepts (see Definition 2). This allows us to exploit another interesting property. Constraints can now be applied over the attributes of the known concepts that compose the target concept, or over the relations between them. Thus, Goci directly includes such constraints in the clauses as literals (see Example 2). Though such constraint literals come from the pre-declared language, they are not directly observed in the input example(s). So an ILP learner will fail to include such literals.

If the human inputs (constraints) are provided upfront before learning, it can be wasteful/irrelevant. More importantly, it places an additional burden on the human. To alleviate this, *our framework explicitly queries for human advice on the relevant constraint literals, which are most useful*. Let 𝕌 be a predefined library of constraint predicates in the language, and let U()∈𝕌 be a relevant constraint literal. Human advice A can be viewed as a preference over the set of relevant constraints {U()}. If $U_{A}$ denotes the preferred set of constraints, then we denote the theory having a preferred constraint literal in the body of a clause as $\tau_{A}$. *(For instance, as per Example* *2* Goci *queries “which of the two sampled constraints* Sub(h_b_, h_s_, 1) & Greater(h_b_, h_s_) *is more useful.” Human could prefer* Sub(*h*_b_, h_s_, 1)*, since it subsumes the other.)* The scoring function now becomes:

(3) $$\begin{array}{l} T^{*}=\underset{T\in \tau }{\text{arg}\text{ min}}\left(-LL\left(T\right)+D\left(T/\theta_{X},X\right)\right)\;:\tau \subseteq \left\{\tau_{A}\right\} \end{array}$$

Thus, we are optimizing the constrained form of the same objective as Equation (1), which aims to prune the search space. This is inspired by advice elicitation approaches (Odom et al., 2015). While our framework can incorporate different forms of advice, we focus on preference over constraints on the logical variables. The formal algorithm, described next, illustrates how we achieve this via an iterative greedy refinement (Figure 3, query-advice loop shown in left).

### 3.3. The Goci Algorithm

Algorithm 1 outlines the Goci framework. It initializes a theory *T*_0_ by variablizing the “*bottom clause”* obtained from *X* and background knowledge [lines 3 and 5]. Then it performs a standard ILP search (described earlier) to propose a candidate theory [line 6]. This is followed by the guided refinement steps, where constraint literals are sampled (parameter tying guides the sampling) and the *human teacher* is queried for preference over them, such that the candidate theory can be modified using preferred constraints [lines 7–9]. The function Ncd() performs the computation of the conceptual distance by first grounding the current modified candidate theory *T*′ with the same parameter values as the input example *X*, then generating grounded plans and finally calculating the normalized compression distance between the plan strings (as shown in Figure 3 and Equation 2) [line 10]. The distance-penalized negative log-likelihood score is estimated and minimized to find the best theory at the current iteration [lines 11–14], which is then used as the initial model in the next iteration. This process is repeated either until convergence (no change in induced theory) or maximum iteration bound (*L*).

**Algorithm 1:** Guided one-shot concept induction.

1: **procedure** Goci(Instance *X*)
2: **Initialize:** Set Iteration ℓ←1
3: **Initialize:** Bootstrap theory $T_{0}\leftarrow X/\theta^{-1}$
4: **repeat**
5: Use *T*_ℓ−1_ as initial model
6: Candidate theory *T*_ℓ_← Search(*T* ∈ τ|*T*_ℓ−1_)
7: **Sample** applicable constraints U∈U
8: $U_{A}\leftarrow$ Query(human,U)
9: $T^{\prime }\leftarrow T_{\ell }⊕U_{A}$ ⊳ $\forall \;U_{A}\in A$
10: $D_{\ell }\left(T^{\prime }/\theta_{X},X\right)\leftarrow \text{NCD}\left(\pi_{T^{\prime }/\theta_{X}},\pi_{X}\right)$
11: Score $S_{\ell }\leftarrow \left(-LL\left(T^{\prime }\right)+D\left(T^{\prime }/\theta_{X},X\right)\right)$
12: **if** *S*_ℓ_ < *S*_ℓ−1_ **then** ⊳ minimize
13: Retain *T*′: Update $T_{\ell }=T^{\prime }$
14: **end if**
15: **until** ℓ ≤ *L* OR *T*_ℓ_ = *T*_ℓ−1_
16: **end procedure**

**Figure 4:**
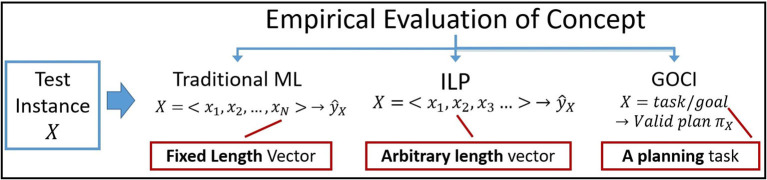
Difference in evaluation of a concept instance across different learning paradigms.

**Connection to plan induction:** Evaluation, both in traditional ML and ILP, generally predicts the value of ŷ_*X*_ for a test instance *X* represented as a fixed (ML) or arbitrary (ILP) length feature vector. In Goci, however, the notion of evaluation of an instance *X* depends on successful construction of a valid/correct plan π_*X*_ (Figure 4). Thus, while learning, most research aim to maximize coverage of positive instances *E*^+^ ($\max P\left({ŷ}_{x}=true|y_{x}\in E^{+}\right)$) and minimize coverage of negatives *E*^−^, [i.e., $\min P\left({ŷ}_{x}=true|y_{x}\in E^{-}\right)$]. Goci evaluates a candidate concept representation by allowing the agent to realize that concept—by computing a valid plan for the goal/task implied by the instance *x*. This is akin to plan induction, since we are learning parameterized plan for realizing the concept as a surrogate for the concept itself. Additionally, planning has long been shown to be conceptually same as logic programming (Preiss and Shai, 1989) and hence induction of logic programs (theories) is the same as plan induction where the examples are trajectories (plan traces) in this case.

### 3.4. Theoretical Analysis

#### 3.4.1. Validity of Distance Metric

NCD δ(*x, y*) between two strings *x* and *y* is provably a valid distance metric (Vitányi et al., 2009): $\delta \left(x,y\right)=\frac{\max K\left(x|y\right),K\left(y|x\right)}{\max K\left(x\right),K\left(y\right)}$, where *K*(*x*) is the Kolmogorov complexity of a string *x* and *K*(*x*|*y*) is the conditional Kolmogorov complexity of *x* given another string *y*. NCD is a computable approximation of the same [*D*(*x, y*) ≈ δ(*x, y*)]. Thus, we just verify if δ is a correct conceptual distance measure. Let *T*_*Y*_ and *T*_*Z*_ be two theories, with same parameterizations (i.e., same heads). Let *T*_*Y*_/θ and *T*_*Z*_/θ be their groundings with identical parameter values θ. Our learned theories are equivalent to planning tasks. Assuming access to a planner Π() which returns *Y* = Π(*T*_*Y*_/θ) and *Z* = Π(*T*_*Z*_/θ), the two plan strings with respect to the instantiations of concepts are represented by *T*_*Y*_ and *T*_*Z*_, respectively.

**Proposition 1** (Valid Conceptual Distance). *Normalized information distance δ*(*Y, Z*) *is a valid and sound conceptual distance measure between T*_*Y*_ *and T*_*Z*_, *that is, δ*(*Y, Z*) = 0 *iff the concepts represented by T*_*Y*_ *and T*_*Z*_ *are equivalent*.

Proof Sketch for Proposition 1: Let *T*_*Y*_ and *T*_*Z*_ be 2 induced consistent first-order Horn clause theories, which may or may not represent the same concept. Let θ be some substitution. Now let *T*_*Y*_/θ and *T*_*Z*_/θ be the grounded theories under the same substitution. This is valid since we are learning horn clause theories with the same head, which indicates the target concept being learned. As explained in the paper, a theory is equivalent to a planning task. We assume access to a planner Π(), and we get plan strings *Y* = Π(*T*_*Y*_/θ) and *Z* = Π(*T*_*Z*_/θ) with respect to the planing tasks *T*_*Y*_/θ and *T*_*Z*_/θ.

Friend et al. (2018) proved that *Conceptual Distance* is the step distance between two consistent theories in a cluster network (𝕋, ⇄, ~), where 𝕋 is the class of consistent theories, ⇄ is the definitional equivalence relation (equivalence over bidirectional concept extensions) and ~ implies symmetry relation. We have shown in the paper that, given the class of concepts we focus on, *a concept is a planning task*.

Let there be a theory T^*^, which represents the optimal generalization of a concept C. If step distance $\langle T_{Y},T^{*}\rangle =0$ in a cluster network and $\langle T_{Z},T^{*}\rangle =0$, then 〈*T*_*Y*_, *T*_*Z*_〉 = 0, that is, they represent the same concept *C* and they are definitionally equivalent *T*_*Y*_ ⇄ *T*_*Z*_. Thus, both *T*_*Y*_/θ and *T*_*Z*_/θ will generate the same set of plans as T^*^, since they will denote the same planning tasks (by structural induction). Thus,

(4) $$\begin{array}{l} T_{Y}⇄T_{Z}\Leftrightarrow \left[\Pi \left(Y\right)\cap \Pi \left(Z\right)=\Pi \left(Y\right)=\Pi \left(Z\right)\right] \end{array}$$

up to equivalence of partial ordering in planning. Let π^*^() be a minimum length plan in a set of plans Π(). Let *y* and *z* be strings indicating plans π^*^(*Y*) and π^*^(*z*) ignoring partial order. If Π(*Y*) = Π(*Z*), then π^*^(*Y*) = π^*^(*z*). Hence, the conditional Kolmogorov complexities *K*(*y*|*z*) and *K*(*z*|*y*) will both be set to 0, if the strings *x* and *y* are equivalent (ignoring partial ordering). This is based on the principle that if they are equivalent, then a Universal prefix-Turing machine will recover one string given the other in 0 steps.

$$∴\frac{max\left(K\left(y|z\right),K\left(z|y\right)\right)}{max\left(K\left(y\right),K\left(z\right)\right)}=0=\delta \left(Y,Z\right)$$

**Proposition 2** (Generalization to Kolmogorov–Smirnov). *In generalized probabilistic logic, following Vitányi (2013), δ*(*Y, Z*) *corresponds to 2-sample* ***Kolmogorov–Smirnov statistic*** *between two random variables T*_*Y*_/*θ and T*_*Z*_/*θ with distributions P*_*T*_*Y*__ *and P*_*T*_*Z*__, *respectively*, [$v\left(T_{Y},T_{Z}\right)={\text{sup}}_{\theta \in F}|F_{T_{Y}}\left(\theta \right)-F_{T_{Z}}\left(\theta \right)|$], *where F*_*T*_() *is the cumulative distribution function for P*_*T*_ *and* ${\text{sup}}_{\theta \in F}$ *is the supremum operator. In a deterministic setting, δ is a special case of the Kolmogorov–Smirnov statistic v*, δ(*Y, Z*) ≼ *v*(*F*_*T*_*Y*__, *F*_*T*_*Z*__).

Proof Sketch for Proposition 2: This can be proved by considering the connection between NID and the distributions induced by the concept classes we are learning. NID is defined as $\delta \left(x,y\right)=\frac{\max K\left(x|y\right),K\left(y|x\right)}{\max K\left(x\right),K\left(y\right)}$, where, *K*(*a*|*b*) is the conditional Kolmogorov complexity of a string *a*, given *b*. There is no provable equivalence between Kolmogorov complexity and traditional notions of probability distributions.

However, if we consider a *reference* universal semi-computable semi-probability mass function **m**(*x*), then there is a provable equivalence −log **m**(*x*) = *K*(*x*) ± *O*(1). Similarly for conditional Kolmogorov complexity, by Conditional Coding Theorem, −log **m**(*y*|*x*) = *K*(*y*|*x*) ± *O*(1) (Vitányi, 2013). By definition,

$$m\left(y|x\right)=\underset{j\geq 1}{\sum }2^{-K\left(j\right)-c_{j}}P_{j}\left(y|x\right)$$

where *c*_*j*_ > 0 are constants and *P*_*j*_(*y*|*x*) is the lower semi-computable conditional. A lower semi-computable semi-probability conditional mass function is based on the string generating complexity of a Universal prefix-Turing machine. Thus, *m*(*y*|*x*) is greater than all the lower semi-computable. Note that our compressed plans are equivalent to a string generated by Universal prefix-Turing machines. The conditional case implies, if a compressed plan string *x* is given as an auxiliary prefix tape, how complex it is to generate compressed string *y* = θ.

Given two grounded theories *T*_*Y*_/θ and *T*_*Z*_/θ, let *P*_*T*_*Y*_/θ_, *P*_*T*_*X*_/θ_ be the respective distributions when learning probabilistic logic rules. Now let us define the semantics of a distribution *P*_*T*/θ_ in our case: *P*_*T*/θ_ = *P*(π(*T*/θ)), that is, distribution over the plan strings, which can be considered as lower semi-computable probability based on coding theory. We know,

(5) $$\begin{array}{l} \underset{j\geq 1}{\sum }2^{-K\left(j\right)-c_{j}}P_{j}\left(y\right)\approx F\left(y|x\right) \end{array}$$

where *F*(*y*) is the cumulative distribution. So, NID δ(*Y, Z*) now becomes, $\delta \left(Y,Z\right)=\frac{max\left(K\left(y|z\right),K\left(z|y\right)\right)}{max\left(K\left(y\right),K\left(z\right)\right)}$ We know that *max*(*K*(*y*), *K*(*z*)) is a normalizer. Thus, δ(*Y, Z*) < *max*(*K*(*y*|*z*), *K*(*z*|*y*))

$$\begin{array}{l} max\left(K(y|z),K(z|y)\right)=max\left(-logm(y|z),-logm(z|y)\right)\\ \;=max\left(-log\frac{m(y,z)}{m(z)},-log\frac{m(y,z)}{m(y)}\right)\\ \;=max(\left[-\log m(y,z)+\log m(z)\right],\\ \;\left[-\log m(y,z)+\log m(y)\right])\\ \text{ Under partial ordering max yields supremum}\\ \;\approx sup\left|\log m(y)-\log m(z)\right|\\ \;\approx sup\left|\log F(y)-\log F(z)\right|\\ \;\approx sup\left|F(y)-F(z)\right|\text{log is monotonic} \end{array}$$

**Significance of Propositions 1 and 2**: Proposition 1 outlines how our proposed NCD-based metric is a valid conceptual distance. It is well understood that the true measure of conceptual distance is not straightforward and is subject to the semantic interpretation of the domain itself. But designing a unique distance metric based on the semantics of every domain limits the generality of any learning system. So NCD acts as a surrogate “conceptual distance.” It is based on the notion that “if two concepts are fundamentally same the complexity of optimal action plans to realize the concepts should also be fundamentally same.” NCD (or NID) essentially measures the difference in generative complexities of two plans. Also note that other types of distances that are limited to a syntactic level such as edit distance (or literal distance) will fail to capture the similarity or diversity between concept representations since the same concept can be represented with more than one theories that may vary in one or more literals.

Proposition 2, on the other hand, proves that our proposed metric is not limited to our specific scenario. It positions our work in the context of known statistical distance metrics and establishes its credibility as a valid solution. It proves how in a nondeterministic setting, that is, probabilistic logic formulation, our proposed metric generalizes to Kolmogorov–Smirnov statistic.

#### 3.4.2. PAC Learnability

PAC analysis of Goci follows from GOLEM for function-free *horn* clause induction (Muggleton and Feng, 1990). Let initial hypothesis space be $H_{0}$ and the final be $H^{*}$ ($s.t.T^{*}\in H^{*}$).

**Proposition 3** (Sample Complexity). *Following Valiant (1984) and Mooney (1994), with probability* (1−δ), the sample complexity of inducing the optimal theory T^*^ *is*:

(6) $$\begin{array}{l} n^{*}=O\left(\frac{1}{\epsilon }\left[d^{L}j^{i}\ln\left(\left(tfm\right)\right)+\ln\left(\frac{1}{\delta }\right)\right]\right) \end{array}$$

*where ϵ is the regret*, n^*^ - *sample complexity of* $H^{*}$, *i is the maximum depth of a variable in a clause and & j is the maximum arity. m* - *number of distinct predicates, t is the number of terms, p is the place and d is the distance of the current revision from the last known consistent theory, and L is the upper bound on the number of refinement steps (iterations)*.

Proof Sketch for Proposition 3: *In our learning setting, the learned theory will always have nonzero uncertainty*. To understand the properties, we build upon the PAC analysis for recursive *rlgg* (Relative Least General Generalization) approach for function-free *Horn* clause learning shown by Muggleton and Feng (1990) in GOELM. With some restrictions, it applies here as well. Let *n*: denote the sample size and H: the hypothesis space. Our approach can be considered as an *rlgg* approach with refinement steps. Note that constraint predicates that refine the clauses are not part of K.

To begin with, we are interested in regret bounds for the initially learned hypothesis by the ILP learner $H_{0}$, before refinement. We know from Valiant (1984), that with probability (1 − δ) the sample complexity *n* for $H_{0}$ is,

(7) $$\begin{array}{l} n\geq \frac{1}{\epsilon }\left(\ln\;\left(\left|H_{0}\right|\right)+\ln\;\left(\frac{1}{\delta }\right)\right) \end{array}$$

where ϵ is the regret. Now, our ILP learner induces *ij*-determinate clauses (Muggleton and Feng, 1990), where *i* is the maximum depth of the clause and *j* is the maximum arity. In our problem setting, it can be proven that $|H_{0}|=O\left({\left(tpm\right)}^{j^{i}}\right)$, where *m* is the number of distinct predicates in the language. *t* is the number of terms, and *p* is the place (for details about place, refer Muggleton and Feng, 1990). Also note that if *j* & *i* is bounded, then *j*^*i*^ ≤ *c*). Mooney (1994) shows that for theory refinement/revision, sample complexity is expressed as,

(8) $$\begin{array}{l} n^{*}=O\left(\frac{1}{\epsilon }\left[d^{k}\ln\;\left(|H_{0}|+d+m\right)+\ln\left(\frac{1}{\delta }\right)\right]\right) \end{array}$$

where distance *d* to be the number of single literal changes in a single refinement step and *k* is the number of refinement/revision iterations. In Algorithm 1, we observe that at each iteration ℓ ≤ *L*, updates are with respect to the preferred constraint predicates $U_{A}\in 𝕌$. Thus, we know that *k* = *L*. Substituting $|H_{0}|={\left(tfm\right)}^{j^{i}}$ and *j*^*i*^ = *c* constant)in Equation (8) and ignoring the additive terms *d* + *m* since (*tfm*)^*j*^^*i*^ >> *d* + *m*, we get,

(9) $$\begin{array}{l} n=O\left(\frac{1}{\epsilon }\left[d^{L}c\ln\;\left(tpm\right)+\ln\;\left(\frac{1}{\delta }\right)\right]\right) \end{array}$$

**Proposition 4** (Refinement Distance). *d is upper bounded by the expected number of literals that can be constructed out of the library of constraint predicates with human advice* ${𝔼}_{\sim A}\left[\left|𝕌\right|\right]$ *and lower bounded by the conceptual distance between theory learned at two consecutive iterations since we adopt a greedy approach. If* $Pr_{A}\left(U\right)$ *denotes the probability of a constraint predicate being preferred, then* |Dℓ-Dℓ-1|≤d≤∑i=12(|𝕌|-1)×PtqPrA(Ui) *where* 2^(|𝕌|−1)^ × ^*t*^*P*_*q*_ *is the maximum possible number of constraint literals and q is the maximum arity of the constraints. In case of only pairwise constraints, q* =2.

Proof Sketch for Proposition 4: The proof is straightforward and hence we present it in brief. In our setting to show that,

(10) $$\begin{array}{l} |D_{\ell }-D_{\ell -1}|\leq d\leq \overset{2^{\left(|𝕌|-1\right)}\times tPq}{\underset{i=1}{\sum }}P_{A}\left(U_{i}\right) \end{array}$$

(where 2^(|𝕌|−1)^ × ^*t*^*P*_*q*_ is the maximum number of constraint literals possible, since 𝕌 is the library of constraint predicates) consider that the number of constraint predicates that can be picked up at any iteration is 2^(|𝕌|−1)^. To form constraint literals, we need to tie arguments to existing logical variables in the current theory. We have defined *t* to be the number of terms in the existing theory. Let *q* be the max arity of a constraint, thus total possible number of constraint literals are 2^(|𝕌|−1)^ × ^*t*^*P*_*q*_. So if the distribution induced on the constraint literals by human advice A be $P_{A}$, then ∑i=12(|𝕌|-1)×PtqPA(Ui) is the expected number of literals added given the advice. Now this is the upper bound of *d*. Again *d* should at least be the conceptual distance between the new theory after constraint addition and the last consistent theory. Note *d* and conceptual distance *D* is not the same. Thus, it is the difference between the NCD of last theory to original example and current updated theory to the original example |*D*_ℓ_ − *D*_ℓ−1_|.

Observe that if at each layer ℓ ≤ *L* we add constraint predicates $U_{\ell }$, then at layer ℓ, $d=|{\left\{U\right\}}^{\ell }|\leq 2^{m}tPq$ (assuming *q* is maximum arity of the constraint predicates). Also, as per our greedy refinement framework, at each layer ℓ, distance new theory 𝕋_ℓ_ should at least be the change in conceptual distance.

**Significance of Propositions 3 and 4:** Propositions 3 and 4 aim to illustrate what the general sample complexity would be for a theory refinement-based RLGG clause learner such as Goci and how the conceptual distance controls the complexity by establishing bounds on the refinement distance. Furthermore, the complexity is also subject to the maximum refinement iterations, which in turn is affected by human guidance. Thus, we establish the theoretical connection between the two dimensions of the contribution of this work.

**Proposition 5** (Advice Complexity). *From Equations (6) and (8), at convergence* ℓ = *L*, *we get* $\frac{n^{*}-|X|}{L}$ *examples, on an average, for a concept* C *to be PAC learnable using* Goci.

The proof is quite straightforward and hence we just discuss the brief idea behind it. Our input is sparse (one or few instances). Goci elicits advice over constraints to acquire additional information. Let |*X*| be the number of input examples. We query the human once at each layer and hence the maximum query budget is *L*. Given that the sample size is |*X*|, each query to the human must acquire information about at least $\frac{n^{*}-|X|}{L}$ examples, on an average, for our a concept C to be PAC learnable using our approach.

## 4. Evaluation

We next aim to answer the following questions explicitly:

**(Q1)** Is Goci effective in “one-shot” concept induction?
**(Q2)** How sample efficient is Goci compared to baselines?
**(Q3)** What is the relative contribution of the novel scoring function versus human guidance toward performance?

Our framework extends a Java version of Aleph (Srinivasan, 2007). We modified the scoring function with NCD penalty computed via a customized SHOP2 planner (Nau et al., 2003). We added constraint sampling and human guidance elicitation iteratively (Algorithm 1).

### 4.1. Experimental Design

We compare Goci with Aleph with no enhancements. We focus on the specific task of “one-shot concept induction,” with a single input example for each of the several types of concepts and report aggregated precision. We consider precision because preference queries are meant to eliminate false positives in our case. To demonstrate general robustness of Goci, beyond one-shot case, we experimented with varying sample sizes for each concept type and show learning curves for the same. We perform an ablation study to show the relative contribution of two important components of Goci: (a) novel scoring metric and (b) human guidance, that is, we compare against two more baselines (*ILP+Score* and *ILP+Guidance*). For every domain, we consider 10 different types of concepts (10 targets) and aggregate results over 5 runs.

Note that human guidance was obtained from distinct human experts for every run. The expertise level of all the advice providers was reasonably at par since they were chosen from the same pool of candidates with zero visibility and knowledge of our proposed framework. However, for all the human advice providers we assumed a basic level of knowledge in geometry or fundamentals of logic and reasoning. Additionally, we also explained each of the experimental domains to the human participants to create a similar level of awareness about the domains among all of them.

#### 4.1.1. Domains

We employ four domains with varying complexity. Note that we have selected the below domains based on multiple considerations. The domain encoding need to be such that target concepts can be learned in a modular fashion (i.e., decomposable). Thus, the first two domains are structure construction domains either spatial (Minecraft) or chemical/molecular (CheBI). Spatial structures are implicitly modular (such as the 𝕃-structure in Figure 1). Chemical entities, molecules/compounds/complexes, are similarly modular as well. The last two domains are fundamentally planning domains. However, they are also compositional in nature, that is, any planning goal is a composition task. For instance, machine structure in “Assembly” domain and cocktails, etc., in “Barman” domain. So these two domains do not just demonstrate learning modular/decomposable concepts but they also illustrate the plan induction feature of Goci.

**Figure 5:**
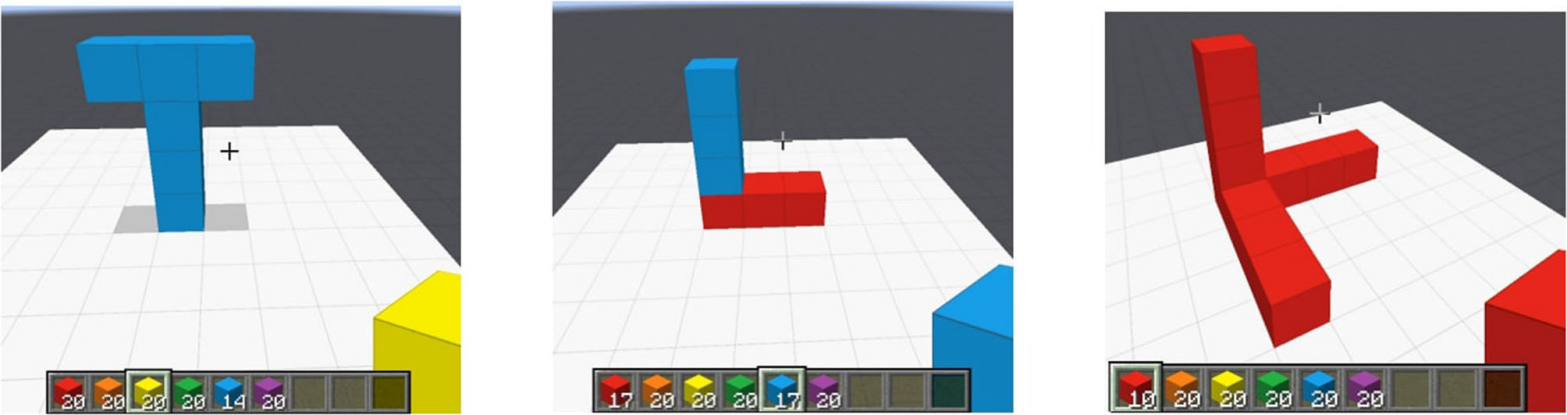
Instances of spatial concepts in Minecraft. **(Left)** Upright Tee, **(Middle)** Upright L, **(Right)** *Orthogonal overlapping Ls*.

1. ***Minecraft (spatial structures):*** The goal is to learn discrete spatial concepts in a customized (Narayan-Chen et al., 2019) Project Malmo platform for Minecraft. Dialogue data in Malmo are available online, and we converted them into a logical representation. All structures are in terms of discrete atomic unit blocks (cubes). Figure 5 shows examples of some spatial structures that Goci was able to learn.
2. ***Chemical Entities of Biological Interest (ChEBI):*** ChEBI (Degtyarenko et al., 2007) is a compound database containing important structural features and activity-based information, for classification of chemicals, such as (1) molecular structure and (2) biological role. We model the Benzene molecule prediction task as molecular-compositional concepts. The data have predicates such as SingleBond, DoubleBond, and HasAtom.
3. ***Assembly (planning domain):*** Assembly is a planning domain, where different mechanical structure concepts are compositions of different parts and resources. Input is a conjunction of ground literals indicating ground plan demonstration (assuming total ordering).
4. ***Barman (planning domain):*** A standard planning domain where a bartender is supposed to follow certain recipes and sequence of techniques to create cocktails. The different cocktails are decomposable concepts in this setting.

### 4.2. Experimental Results

**[Effective One-shot (Q1)]** Table 1 shows the performance of Goci on one-shot concept learning tasks as compared to standard ILP. Goci significantly outperforms ILP across all domains answering (Q1) affirmatively. Also, note that Goci is very “query” efficient as observed from the small average number of queries posed in the case of each domain. Note that in the case of CheBI, the number of queries is the highest among all the domains. This can be attributed to that fact that CheBI is a domain, which requires a certain degree of understanding of fundamental chemistry (chemical bonds and their types, molecules, atoms, etc.). Thus, some of the human participants required more iterations (consequently more queries) to converge to the most relevant set of constraint literals, given the difference of their prior understanding of school chemistry.

**Table 1:** Results for one-shot concept learning.

**Domain**	**Approach**	**Avg. precision**	**#Queries**
Minecraft	Goci	0.85	5.5 ± 3
	ILP	0.35	–
Assembly	Goci	0.65	16.5 ± 4
	ILP	0.2	–
ChEBI	Goci	0.615	13.1 ± 2.13
	ILP	0.45	–
Barman	Goci	0.7	10.5 ± 5.4
	ILP	0.51	–

Query efficiency is an important consideration in any learning paradigm that leverages human guidance, since controlling the cognitive load on the human expert is critical. So, in general, the observed average query numbers being reasonably low across all domains corroborates our theoretical advice complexity (section 3.4.2).

**Figure 6:**
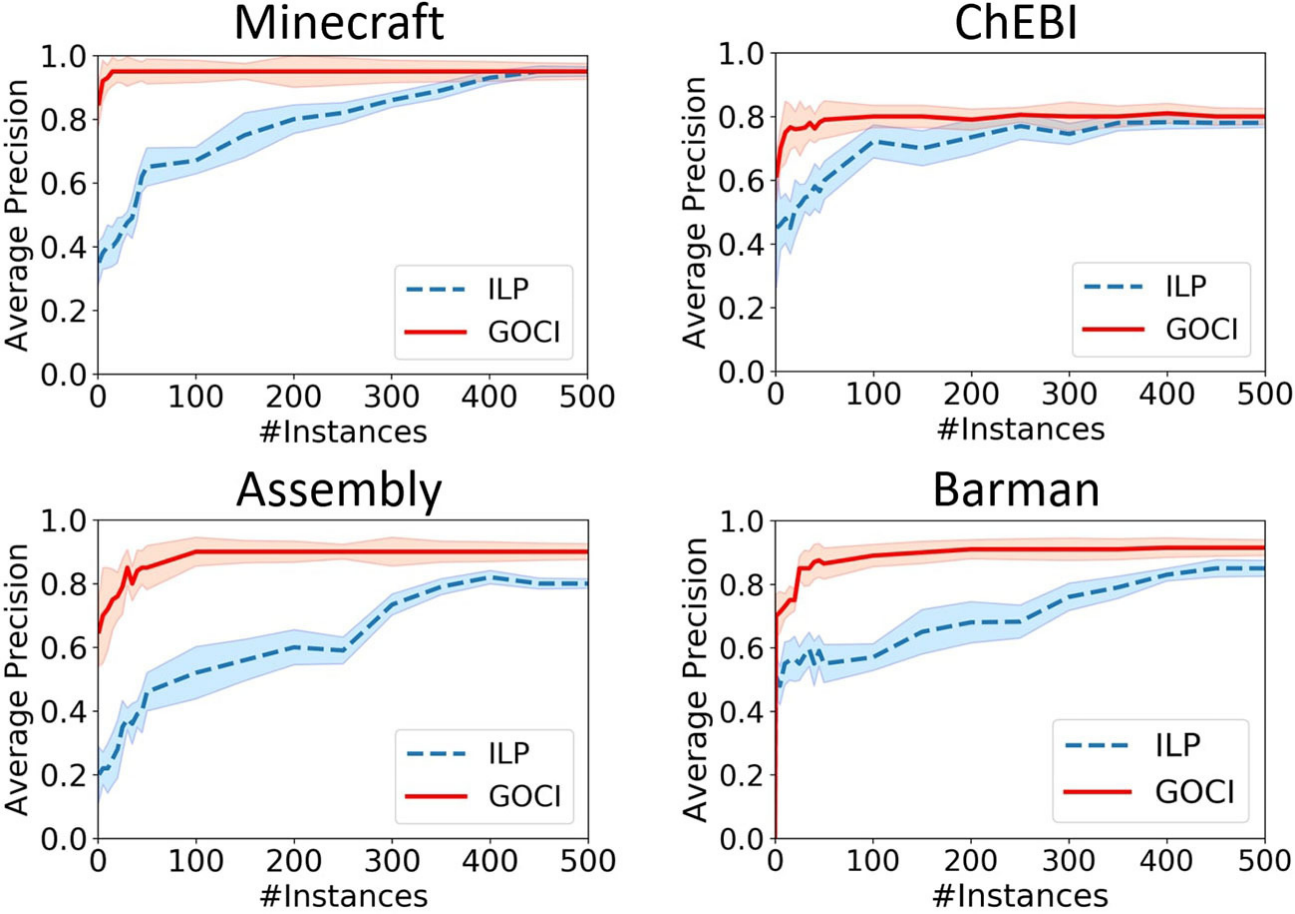
Learning curves for varying sample size to compare the sample efficiency of Guided One-shot Concept Induction (Goci) and inductive logic program (ILP). Top two plots are with respect to structural composition domains-Minecraft & ChEBI and the bottom two are for planning domains: Assembly and Barman (best viewed in color).

**[Sample Efficiency (Q2)]** In Figure 6, we observe that Goci converges within significantly smaller sample size across all domains, thus, supporting our theoretical claims in section 3.4. In ChEBI, though, quality of planner encoding might explain mildly lower precision yet Goci does perform significantly better than vanilla ILP learner. In ChEBI, we see that the sample efficiency is not vastly distinct. One of the possible reasons could be the sub-optimal encoding of the planning domain language, which is necessary for NCD computation, for this task. If we can improve the planner setup for this domain, then we will likely be able to observe enhanced performance.

**Figure 7:**
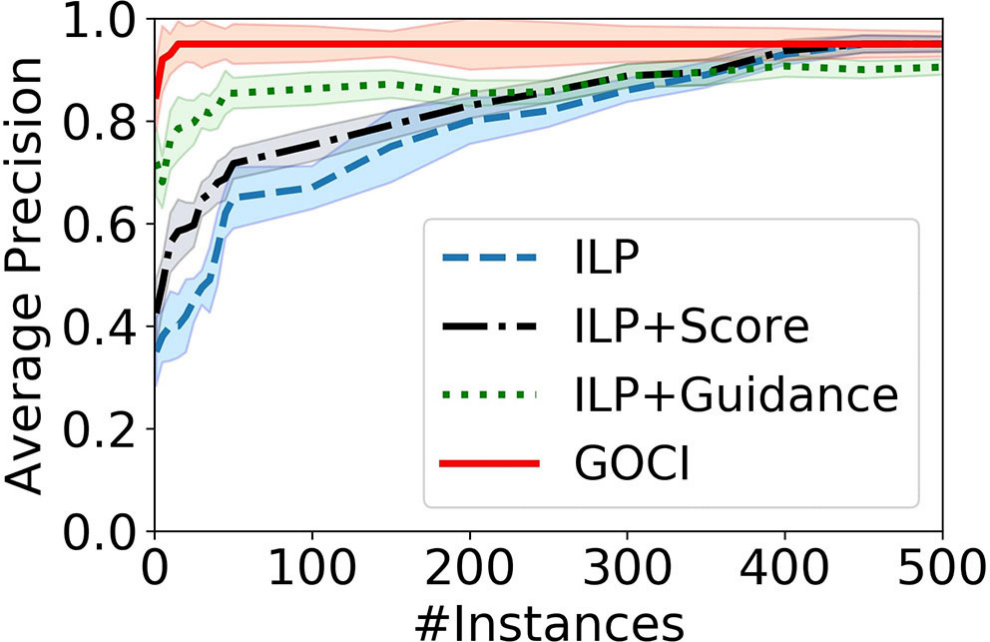
Results of ablation study on Minecraft domain. Relative contribution of our distance-penalized score vs. human guidance.

**[Relative contribution (Q3)]** Figure 7 validates our intuition that both components (scoring function and human-guidance) together make Goci a robust one-shot (sample-efficient) concept induction framework. Though human guidance, alone, is able to enhance the performance of a vanilla ILP learner in sparse samples, yet it is not sufficient for optimal performance. In contrast, although the advantage of our novel distance-penalized scoring metric is marginal in sparse samples, it is essential for optimal performance at convergence.

## 5. Discussion

The most important conclusion from the experiments is that when available, the guidance along with the novel score leads to a jump-start, better slope and in some cases, asymptotically sample efficient with a fraction of the number of instances needed than merely learning from data.

Another important aspect to note here is that our experimental setup did not attempt to ensure in any way that the quality of guidance provided by the human participants is optimal. The formulation of the objective function, itself, in Goci is designed to handle sub-optimal human advice implicitly in a seamless manner. The two primary features in the design that make Goci robust to advice quality are as follows:

1. As explained earlier and shown in Equation (3), human advice and conceptual distance deal with two distinct aspects of the search process. Human advice controls the size and nature of the search space while conceptual distance ensures the quality of the candidates. Advice and distance have a balancing effect on each other, and thus, it is our novel conceptual distance that makes Goci robust to bad advice.
2. Also, the nature of human advice in our setting is of choosing the most useful set of “constraint predicates” among the set of candidate constraints. Now the candidates are generated by Goci in a conservative fashion selecting only the ones that are logically valid for the theory learned at the current iteration of revision. Thus, human experts have very little option of choosing an invalid or extremely unlikely constraint predicate.

Our ablation study in Figure 7 also supports our analysis. On closer inspection, we see that it is due to our novel distance penalized scoring function (ILP+Score) that ensures convergence to an optimal solution. Human advice (ILP+Guidance) contributes to sample efficiency.

## 6. Conclusions

We developed a human-in-the-loop one-shot concept learning framework in which the agent learns a generalized representation of a concept as FOL rules, from a single (few) positive example(s). We make two specific contributions: deriving a new distance measure between concepts and allowing for richer human inputs than mere labels, solicited actively by the agent. Our theoretical and experimental analyses show the promise of Goci method. An exhaustive evaluation involving richer human inputs including varying levels of expertise and analyzing our claim that learning performance of Goci is robust to expertise levels (which should only affect query efficiency) is an immediate future research objective. Integration with hierarchy learning also remains an interesting direction for future research.

## Data Availability Statement

All datasets generated for this study are included in the article/supplementary material.

## Author Contributions

MD and SN contributed equally to the ideation. MD and NR led the empirical evaluation. MD, NR, SN, and JD contributed nearly equally to the manuscript preparation. All authors contributed to the article and approved the submitted version.

## Conflict of Interest

MD was employed by the company Samsung R&D Institute India - Bangalore. The remaining authors declare that the research was conducted in the absence of any commercial or financial relationships that could be construed as a potential conflict of interest.

## Footnotes

^1^Our algorithm can learn from one (few) example(s). We specify the number of examples in our evaluations.
